# Octahedral Tilt-Driven Phase Transitions in BaZrS_3_ Chalcogenide Perovskite

**DOI:** 10.1021/acs.jpclett.4c03517

**Published:** 2025-02-19

**Authors:** Prakriti Kayastha, Erik Fransson, Paul Erhart, Lucy Whalley

**Affiliations:** † Department of Mathematics, Physics and Electrical Engineering, Northumbria University, Newcastle upon Tyne NE1 8QH, United Kingdom; ‡ Department of Physics, Chalmers University of Technology, SE-41296 Gothenburg, Sweden

## Abstract

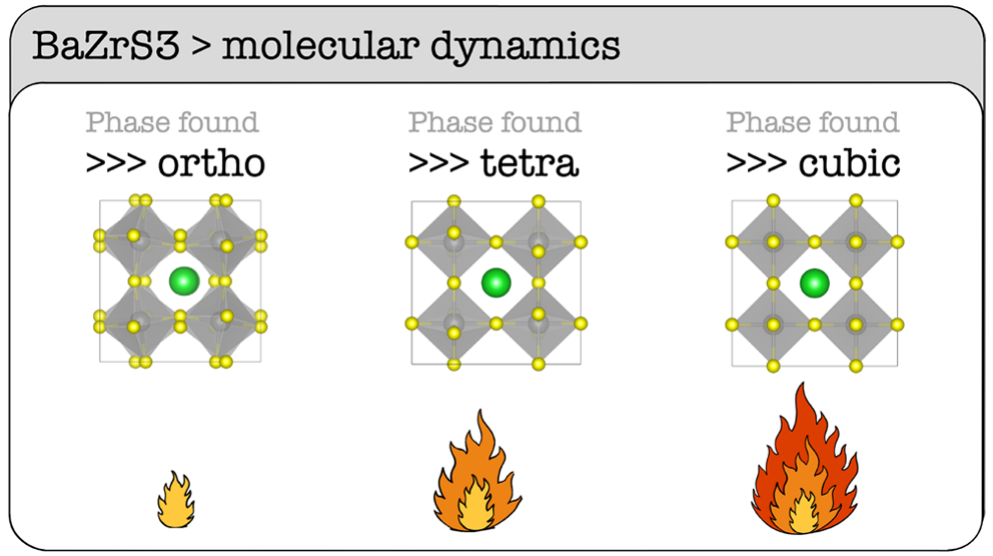

Chalcogenide perovskites are lead-free materials for potential photovoltaic or thermoelectric applications. BaZrS_3_ is the most-studied member of this family due to its superior thermal and chemical stability, desirable optoelectronic properties, and low thermal conductivity. Phase transitions in BaZrS_3_ remain underexplored in the literature, as most experimental characterizations of this material have been performed at ambient conditions where the orthorhombic *Pnma* phase is reported to be stable. In this work, we study the dynamics of BaZrS_3_ across a range of temperatures and pressures using an accurate machine learning interatomic potential trained with data from hybrid density functional theory calculations. At 0 Pa, we find a first-order phase transition from the orthorhombic to tetragonal *I*4/*mcm* phase at 610 K, and a second-order transition from the tetragonal to the cubic *Pm*3̅*m* phase at 880 K. The tetragonal phase is stable over a larger temperature range at higher pressures. To confirm the validity of our model we compare our results with a range of published experimental data and report a prediction for the X-ray diffraction pattern as a function of temperature.

Chalcogenide perovskites have gained relevance as lead-free photovoltaic absorber materials as they exhibit strong light absorption and dielectric screening alongside desirable defect properties.^1−9^ BaZrS_3_ is the most studied member of this family with research efforts towards material synthesis at moderate temperatures, band gap engineering, and a proof-of-concept solar cell.^10−19^ BaZrS_3_ has also been explored as a potential thermoelectric material as it displays fast electronic transport coupled with low thermal conductivity, leading to record-high *zT* values among reported halide and chalcogenide perovskite materials.^20−22^

While BaZrS_3_ is reported to retain stability in a perovskite structure above 1000 K, the vast majority of materials characterization is carried out at ambient conditions.^10,11,23−25^ At room temperature, the consensus is that BaZrS_3_ is stable in an orthorhombic *Pnma* perovskite structure, as confirmed by both experimental and computational studies.^13,21,26−30^ Above this temperature, the picture is less clear. Recent temperature-dependent X-ray diffraction (XRD) measurements show a discontinuous change in the lattice parameters at high temperature, indicating a first-order phase transition.^23,31,32^ However a temperature-dependent Raman spectroscopy study does not confirm this observation,^33^ likely due to the structural and dynamic similarity of perovskite phases coupled with significant thermal broadening.

Many ABX_3_ perovskites undergo tilt-driven phase transitions to form lower-symmetry polymorphs with antiferrodistortive displacement patterns.^34^ The prototypical high-temperature perovskite phase is a cubic structure. As the temperature is reduced, lower-symmetry tetragonal and orthorhombic perovskite phases can be formed through tilting of the BX_6_ octahedra.^35,36^ Therefore, it is likely that there are transitions to higher symmetry phases at temperatures above ambient for BaZrS_3_. Phase transitions may occur before reaching the elevated temperatures required for BaZrS_3_ synthesis (>850 K).^13^ If this is the case it follows that samples grown at high temperature may include mixtures of polymorphs, as has been observed for halide perovskites.^37,38^ A phase transition within the operating temperature range for thermoelectric generators (400 to 1100 K) is also possible. An understanding of the exact BaZrS_3_ perovskite structure is important as even small changes to structure can impact key functional properties including the band gap.^39,40^ Anharmonic dynamics are also crucial for quantitative predictions of electron–phonon coupling and related optical properties.

In this work, we use molecular dynamics (MD) to sample the anharmonic free energy surface and simulate the finite-temperature dynamics of the BaZrS_3_ perovskite. We accelerate the calculation of free energies, atomic forces, and stress tensors necessary for MD by constructing a machine learning interatomic potential with training data from density functional theory (DFT) calculations using a hybrid exchange-correlation functional. We apply known group-subgroup relationships to systematically identify which octahedral tilt patterns can be accessed during phase transitions. We identify two phase transitions in BaZrS_3_ at 0 Pa: a first-order orthorhombic *Pnma* to tetragonal *I*4/*mcm* transition at 610 K and a second-order tetragonal *I*4/*mcm* to cubic *Pm*3̅*m* phase transition at 880 K. We construct a phase diagram for BaZrS_3_ across a pressure and temperature range of −4 to 10 GPa and 0 K 1200 K, respectively. Lastly, we predict the Raman spectra and temperature-dependent XRD patterns, and compare our predictions against published experimental data.

## Methods

A machine learning interatomic potential was constructed using the neuroevolution potential (NEP) method implemented in the gpumd package.^41^ The ase and calorine packages were used to prepare the training structures, set up MD simulations, and postprocess the results.^42,43^ The training set consists of 1187 perovskite structures. This includes cubic, tetragonal, and orthorhombic phases with applied strain or small random displacements, all 15 Glazer-tilt structures,^44,45^ and snapshots from NPT MD simulations. The training set also contains 92 Ruddlesden–Popper structures, which will be the subject of a future publication. Symmetry-constrained geometry relaxations as implemented in ase were performed until the maximal force component was below 10^–3^ eV/Å.^42^ DFT calculations were performed using the fhi-aims code and the HSE06 exchange-correlation functional.^46,47^ The root mean squared training errors were 1.8 meV/atom, 72.2 meV/Å, and 28.9 meV/atom for formation energies, atomic forces, and virials, respectively; see Figure S1 for the loss curves and Figure S2 for the parity plots. Harmonic phonon dispersions were evaluated using the phonopy package with 2 × 2 × 2 supercells and 0.01 Å displacements.^48^ For a comparison of the NEP-calculated and DFT-calculated harmonic phonon dispersions see Figures S3–S5.

Heating and cooling simulations with supercells of 40 960 atoms were run in the NPT ensemble in the temperature range of 0 to 1200 K and a pressure range of −4 to 10 GPa using a time step of 1 fs. To identify the symmetry group formed, atomic displacements were projected onto the octahedral-tilt phonon eigenvectors of the cubic structure, as outlined in ref (49). Mode projections amplitudes for the NEP-relaxed and DFT-relaxed structures are given in Tables S2 and S1, respectively. Free energy calculations were carried out using thermodynamic integration (TI) with an Einstein crystal as reference Hamiltonian.^50^ To calculate the XRD pattern *I*(**θ**) the dynasor package was used to postprocess NVT MD simulations.^51^ For more computational details see the Supporting Information.

**Figure 1:**
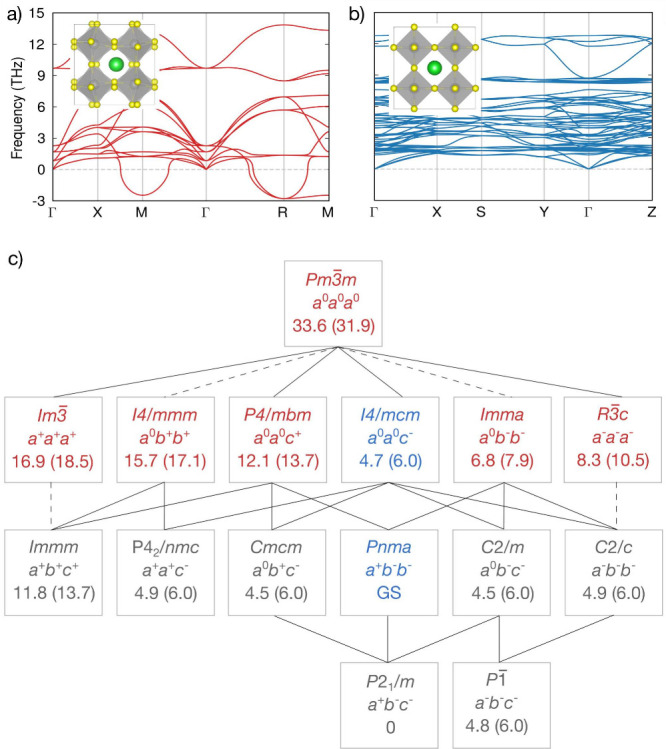
DFT-calculated crystal and phonon band structures of the (a) orthorhombic *Pnma* and (b) cubic *Pm*3̅*m* phases. Green, gray, and yellow spheres represent Ba, Zr, and S atoms, respectively. (c) Group-subgroup relationships and 0 K formation energies. The space group, Glazer notation, and formation energy are specified for each phase accessible through octahedral tilting. The formation energies (in meV/atom) with respect to the ground-state (GS) *Pnma* phase obtained from DFT and NEP model calculations (in parentheses) are reported in the bottom rows. Connecting lines indicate group-subgroup relationships. Dashed lines indicate transitions that must be first order according to Landau theory.^45^ Red text denotes the presence of phonon modes with an imaginary frequency, indicating dynamic instability at 0 K. Blue text denotes dynamic stability. Gray text denotes that the phase is symmetrically equivalent to a supergroup structure after relaxation. There may still be a small discrepancy in formation energy, which is discussed in the Supporting Information. Figure adapted from Howard and Stokes.^45^

In Figure 1a and Figure 1b we plot the harmonic phonon dispersions and crystal structures of BaZrS_3_ in the cubic *Pm*3̅*m* phase and experimentally observed *Pnma* phase. The aristotype cubic *Pm*3̅*m* phase is the simplest perovskite form. However, perovskites often adopt lower-symmetry, distorted noncubic phases.^52^ Distortions in the cubic perovskite give rise to a wide range of structures which can be classified into three categories: (i) BX_6_ octahedral tilting; (ii) distortions of the BX_6_ octahedra; and (iii) B-site cation displacements.^53,54^ Octahedral tilting leads to 15 possible space groups as identified by Glazer.^44^

BaZrS_3_ in the cubic *Pm*3̅*m* phase (*a*^0^*a*^0^*a*^0^ in Glazer notation) is dynamically unstable indicating the presence of a lower-symmetry stable structure at 0 K.^55^ The imaginary phonon modes at the M point of the Brillouin zone correspond to in-phase (^+^) tilting of the ZrS_6_ octahedra and are described with the irrep M_2_^+^ (for a unit cell with an origin at the Ba-site).^56^ The imaginary modes at the R point correspond to out-of-phase (^−^) tilting and have irrep R_5_^–^. Both modes are triply degenerate. Distortions along one M-mode and two perpendicular R-modes result in a dynamically stable orthorhombic *Pnma* phase (*a*^+^*b*^–^*b*^–^). The dynamic and energetic stability of the orthorhombic phase at 0 K is in agreement with previous experimental and DFT studies reporting it to be stable at low temperatures.^57−59^

Distortions along linear combinations of the M- and R-modes result in 15 unique space groups. We display the group-subgroup relationships and 0 K formation energies for BaZrS_3_ in Figure 1c. As expected, *Pm*3̅*m* is the highest energy phase relative to the *Pnma* ground state. *I*4/*mcm* is 4.7 meV/atom above the ground state, indicating that it may form as a higher temperature phase. The comprehensive mapping across all possible structures ensures that we include all octahedral tilt patterns that might be formed at high temperatures in our training data for the machine learning interatomic potential.

Connecting lines in Figure 1c indicate group-subgroup relationships between structures. In Landau theory, this relationship is necessary (but not sufficient) for structures connected through second-order (continuous) phase transitions.^60^ Dashed lines indicate that, despite sharing a group-subgroup relationship, the phase transition must be first-order (discontinuous) in Landau theory.^61^

**Figure 2:**
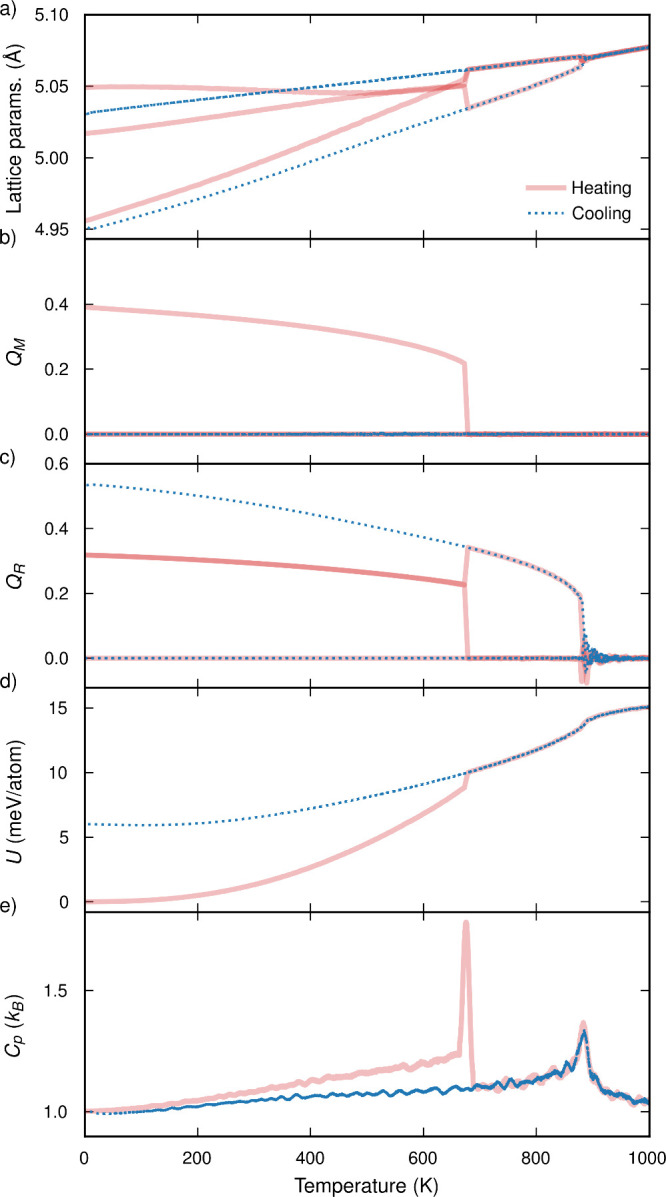
Properties of the BaZrS_3_ perovskite from cooling (blue) and heating (red) simulations: (a) pseudocubic lattice parameters; (b) M-mode and (c) R-mode amplitudes (*Q*_M_, *Q*_R_); (d) energies (*U*); (e) heat capacities (*C*_*p*_). Energies are shown relative to the *Pnma* ground-state energy at 0 K with the equipartition energy (*k*_B_*T*) subtracted. The heat capacity is obtained by calculating the numerical derivative of the energy with respect to temperature, *C*_*p*_ = d*U*/d*T*, and is reported per degree of freedom in the system. The simulation time scale is 200 ns. All quantities are averaged over a time period of 0.8 ns.

In Figure 2, we plot properties observed and derived from MD simulations spanning 0 to 1200 K and with no applied pressure. When heating the low-temperature *Pnma* structure there are phase transitions at 650 and 880 K. The transition at 650 K is accompanied by a discontinuous and sharp change in lattice parameters (Figure 2a). Two of the lattice parameters become equal, indicating an orthorhombic to tetragonal transition. In contrast, the transition at 880 K is gradual and continuous. All three lattice parameters become equal, indicating a tetragonal-to-cubic transition.

In Figure 2b,c we show projections of the M and R phonon modes on structures sampled from the simulation. From 0 to 650 K one M-mode and two R-modes are active (have a non-zero amplitude). This tilt pattern is described by *a*^+^*b*^–^*b*^–^ in Glazer notation and corresponds to a structure in the *Pnma* space group (see Figure 1 and the associated discussion). From 650 K to 880, only one R-mode is activated corresponding to the tetragonal *I*4/*mcm* phase (*a*^0^*a*^0^*c*^–^). Above 900 K, no modes are activated indicating the existence of a cubic *Pm*3̅*m* phase (*a*^0^*a*^0^*a*^0^).

A sharp discontinuity is observed in energy (Figure 2d) and heat capacity (Figure 2e) at 650 K. The 1 meV/atom energy change is the latent heat associated with a first-order phase transition and is comparable to that observed in other perovskites.^35,36^ At 880 K a continuous change in energy is observed, typical of second-order phase transitions, resulting in a broader, less pronounced peak in the heat capacity. We conclude that there is a first-order *Pnma*-to-*I*4/*mcm* transition at 650 K, and a second-order *I*4/*mcm*-to-*Pm*3̅*m* transition at 880 K. These observations are consistent with the group-subgroup analysis presented in Figure 1. The *Pnma* phase does not share a group-subgroup relationship with *I*4/*mcm*, necessitating a first-order phase transition. In contrast, is a subgroup of the *Pm*3̅*m* phase, so can be accessed through a second-order transition.

In the cooling runs, we start from the high-temperature *Pm*3̅*m* structure and reproduce the heating behavior for the second-order transition at 880 K. Significant hysteresis is observed for the first-order phase transition at 650 K as the system cannot overcome the free energy barrier required to form the orthorhombic phase. Due to the stochastic nature of MD simulations, we do recover the orthorhombic phase in some of the cooling runs (Figure S6). Hysteresis in simulations describing a first-order transition has been observed and discussed in previous studies.^35,36,62^

**Figure 3:**
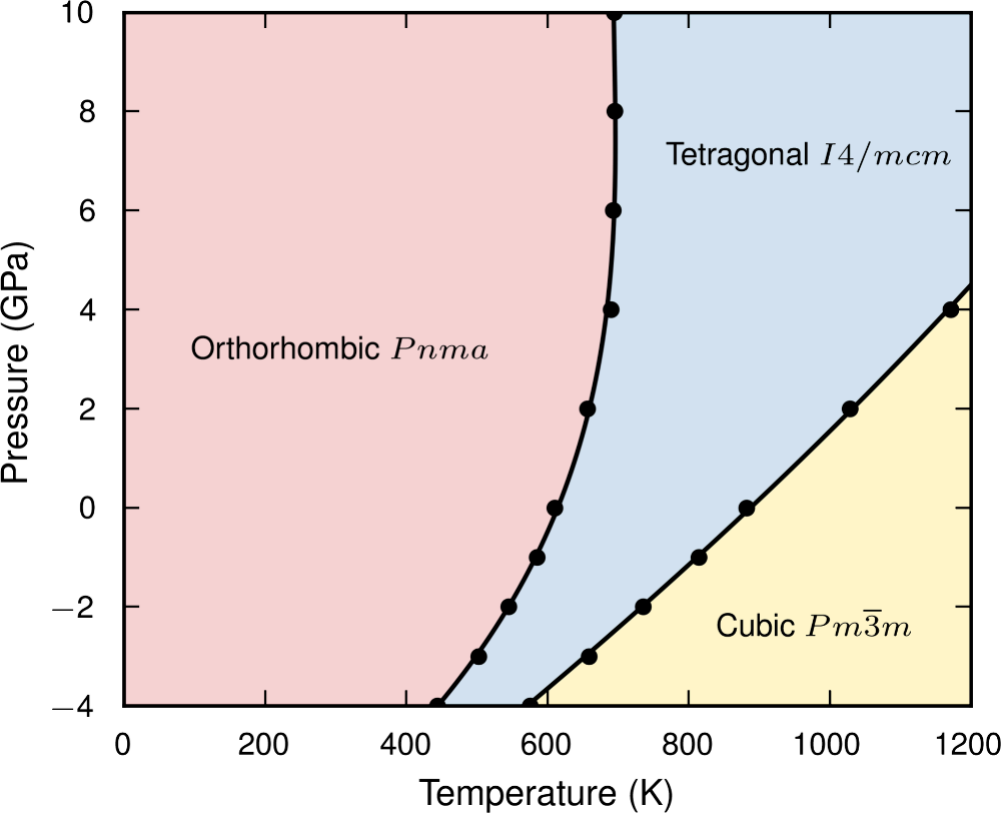
Phase diagram of BaZrS_3_ as a function of pressure and temperature. To predict the first-order *Pnma*-to-*I*4/*mcm* phase transition temperatures we use thermodynamic integration to calculate free energies. The second-order *I*4/*mcm*-to-*Pm*3̅*m* phase transition temperatures are calculated from heating runs (Figure 2).

In many materials transitions to new phases can be induced through applied pressure or strain. In Figure 3, we present the BaZrS_3_ pressure–temperature phase diagram across −4 to 10 GPa and 0 to 1000 K. Negative pressures correspond to triaxial tensile strain. While this is difficult to realize experimentally, tensile strain in a plane can be produced through coherent interface formation with a suitably matched substrate.^63^ As such, Figure 3 indicates the range of polymorphs which might be accessed through interface engineering in a device stack. To accurately predict the first-order *Pnma*-to-*I*4/*mcm* phase transition temperatures we use thermodynamic integration to calculate free energies. This still describes the full anharmonicity of the material but avoids the kinetic limitations of a cooling or heating simulation which must overcome the first-order transition barrier.^36^

The higher symmetry phases are stabilized with increasing temperature or decreasing pressure. This indicates that the ZrS_6_ octahedra are relatively rigid, with volume expansion driven through decreased octahedral tilting;^64^ see the discussion on perovskite bond compressibility in the SI. At zero pressure the *Pnma*-to-*I*4/*mcm* phase transition temperature is 610 K. For comparison, the phase transition temperature in the harmonic approximation is 243 K (Figure S8). Above 4 GPa, the transition temperature saturates at 690 K. Below 400 K there are no structural changes between −4 to 10 GPa. This is in agreement with a previous DFT study at 0 K and Raman measurements across the pressure range 0 to 8.9 GPa.^28,65^

Our simulations show that BaZrS_3_ forms in the *Pnma* structure at room temperature. This observation is supported by experimental characterization in ambient conditions and computational predictions at 0 K.^2,19,28,66^ A recent computational study predicts that the polar *Pna*2_1_ phase is 0.05 meV per formula unit more stable than *Pnma* at 0 K and 0 GPa.^67^ This instability has been observed across a variety of oxide perovskites in the orthorhombic *Pnma* phase.^68^ For BaZrS_3_ the small 0.05 meV energy difference follows small differences in atomic coordinates, with an extremely tight symmetry tolerance of 0.003 Å required to differentiate between the phases.

A multimodal study combining synchrotron XRD, Raman spectroscopy, optical measurements and thermal analysis as a function of temperature identified three polymorphs when BaZrS_3_ is heated in air.^32^ Rietveld analysis of the synchrotron powder XRD patterns showed the *I*4/*mcm* phase to be stable above 770 K and the *Pnma* phase to be stable below 570 K. From 570 to 770 K indirect observations suggest that the orthorhombic *Cmcm* space group co-exists as a minority phase. Despite including the *Cmcm* phase in our training data, our simulations do not predict *Cmcm* as a stable intermediate phase. In fact, at 0 K our DFT calculations show that this phase is kinetically unstable and relaxes to the higher symmetry *I*4/*mcm* phase (Table S1).

In a separate study from Bystrický et al., temperature-dependent XRD data from a non-synchrotron source also indicated an orthorhombic-to-tetragonal phase transition at 770 K.^23,31^ Full structure refinements were not presented and the measurements were partially obstructed through oxidation. According to the analysis of that data above 770 K, the two unique lattice parameters converge,^23^ which we also observe while approaching the second-order *I*4/*mcm*-to-*Pm*3̅*m* transition in our MD simulation (Figure 2a).

**Figure 4:**
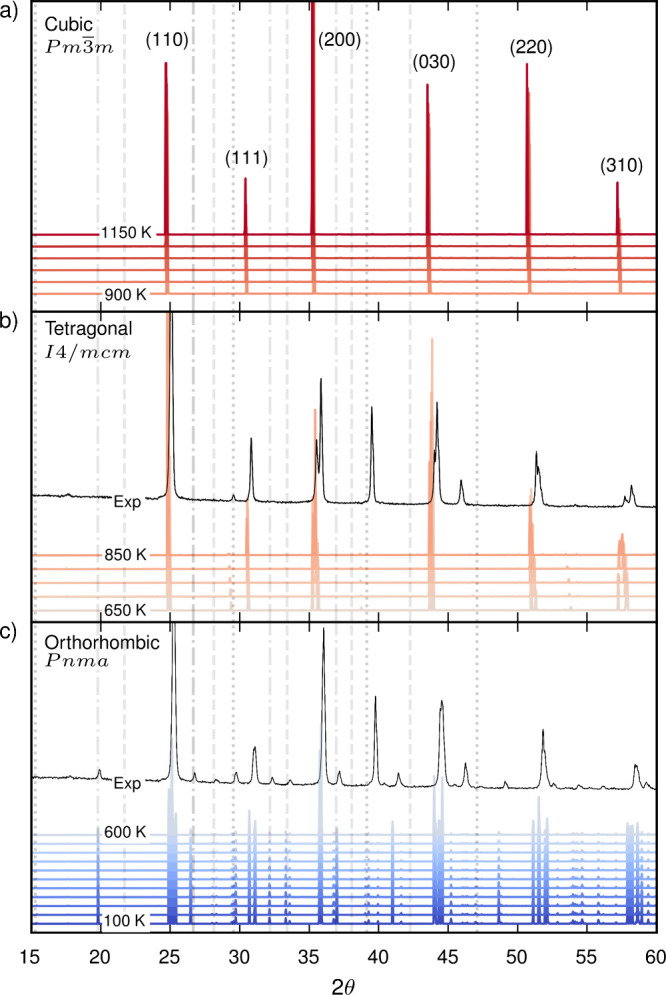
X-ray diffraction (XRD) pattern evaluated for three BaZrS_3_ polymorphs. The temperature ranges from 100 to 1150 K in intervals of 50 K. All simulations are at 0 Pa. A Cu Kα value of 1.5406 Å was used for the *q* to θ conversion. Cubic *Pm*3̅*m* peaks are indexed. Superlattice peaks at half integer planes up to the third Brillouin zone are indicated with vertical lines. The R-, M-, and X-point distortions are represented with dotted, dashed, and dash-dotted lines, respectively. For comparison, Figure S11 displays the static structure factor up to the fifth Brillouin zone. Experimental XRD data for the orthorhombic (303 K) and tetragonal (923 K) phases from ref (31) are plotted in black. The peaks in the experimental data at 39° and 46° are associated with the Pt strip used for heating the sample.

We present a prediction of the temperature-dependent XRD pattern in Figure 4. At high temperature, the characteristic peaks of a cubic perovskite are clearly identified (Figure 4a). Below 900 K, we observe peak splittings of the cubic diffraction lines. From 650 to 900 K the largest splitting corresponds to a *h00* reflection (200), and the *hhh* reflection (111) remains a singlet, indicating a tetragonal distortion.^54^

Experimental XRD data from Bystrický et al. is displayed in Figure 4b for comparison against our predictions.^31^ The structure was refined in the tetragonal space group *I*4_1_/*acd* which, to the best of our knowledge, has not been previously reported for an ABX_3_ perovskite. We find good agreement between the experimental results and our prediction for the higher-symmetry *I*4/*mcm* phase. R-mode activation produces a doubling of the unit cell along one axis and the appearance of a superlattice peak at a half-integer plane. This can be seen in both the experimental and simulation data at 2θ ≈ 29°.

Below 600 K, an abrupt change is observed due to the first-order phase transition into the orthorhombic phase (Figure 4c). M-point distortions result in the appearance of additional superlattice peaks at 27° and 33° alongside further peak splitting. Superlattice peaks associated with X-mode distortions also appear at 20°, 26°, 32° and 37°. The same behavior is observed in the experimental XRD data measured at 303 K.^31^ Decomposition of the XRD intensity (Figure S13) shows that some X-point peaks are associated with off-centering of the Ba species. A-site cation off-centering is frequently observed when R-point and M-point distortions operate in tandem.^39^

Transitions between structurally similar perovskite phases are not always discernible in Raman spectra, as the peak splitting can be less than the peak broadening resulting from thermal fluctuations or higher-order scattering.^69−71^ The spectral energy densities of the *I*4/*mcm* and *Pm*3̅*m* phases demonstrate that there is considerable phonon broadening at elevated temperatures (Figure S7). While Ye et al. reported that there is no indication of a first-order phase transition between 10 to 875 K,^33^ Jaiswal et al. found the number of Raman peaks to decrease with increasing temperature, indicative of a phase transition to a higher-symmetry structure.^32^ Our simulated Raman spectra in Figure S9 and Figure S10 demonstrates that there is significant peak overlap between the *Pnma* and *I*4/*mcm* phases. Our spectra also reproduces the two most pronounced changes with temperature from Jaiswel et al.: removal of the *A*_*g*_^6^ peak and a significant shift in the *B*_*g*_^6^ peak position.

Experimental characterization of the high-temperature *Pm*3̅*m* phase is hindered by oxidation which leads to the formation of BaSO_4_, ZrO_2_ and SO_2_. Differential scanning calorimetry and thermogravimetric analysis show that BaZrS_3_ is stable in air up to 920 K, with complete conversion to the oxidized products at 970 K.^32,66^

In conclusion, chalcogenide perovskites, in particular BaZr, show great potential for applications in optoelectronic and thermoelectric technologies. However, several aspects of fundamental material behavior, including polymorphic phase transitions, have not previously been explored in detail. In addition, experimental characterisations of the structure through Raman spectroscopy and XRD give conflicting results. We address this problem by developing a machine learning interatomic potential for BaZrS_3_ trained on hybrid DFT calculations. This is used to run high-accuracy MD simulations across a wide range of temperatures and pressures.

The structural and thermodynamic properties derived from heating simulations reveal a series of transitions from orthorhombic *Pnma*-to-tetragonal *I*4/*mcm*-to-cubic *Pm*3̅*m* with increasing temperature. While this sequence of structures—from the low-symmetry *Pnma* phase to the high-symmetry *Pm*3̅*m* phase—is commonly observed in perovskite materials, to the best of our knowledge this is the first report for BaZrS_3_. There is no evidence for additional transitions beyond these before melting. The predicted character of each transition—first-order *Pnma*-to-*I*4/*mcm* and second-order *I*4/*mcm*-to-*Pm*3̅*m*—is in agreement with those allowed by group-subgroup relationships.

Both phase transitions occur above 600 K, which agrees with experimental characterization showing BaZrS_3_ is stable in the orthorhombic *Pnma* phase at ambient temperature and pressure. In addition, the calculated Raman spectra and temperature-dependent XRD patterns align well with experimental data, supporting our prediction of an orthorhombic-to-tetragonal phase transition and validating our overall approach. The second-order transition at 880 K is more difficult to characterize due to the concurrent high-temperature oxidation processes; further experimental studies in an inert atmosphere are required for confirmation.

It is possible that BaZrS_3_ samples grown at high temperature may include mixtures of polymorphs. Future work might more fully consider polymorph mixing, alongside the impact of octahedral tilting on the thermal and optoelectronic properties of BaZrS_3_. We note that the formation of ternary Ruddlesden–Popper phases Ba_*n*+1_Zr_*n*_S_3*n*+1_ has been considered elsewhere in the literature.^30,72,73^ When formed these are likely to have a greater impact on material properties through disruption of the 3D octahedral framework.

## Data Availability

The NEP models generated in this study are openly available via Zenodo at https://dx.doi.org/10.5281/zenodo.14229468. The DFT output data has been uploaded to the NOMAD repository and is available at https://dx.doi.org/10.17172/NOMAD/2024.11.25-2. A separate repository is also hosted at https://github.com/NU-CEM/2024_BaZrS3_Phase_Transitions with Python code available to reproduce the figures and analysis.

## Supporting Information Available

Methods and NEP model validation, phonon mode projections for the Glazer tilt structures, results from a cooling run at −1 GPa, temperature-dependent phonon dispersions from molecular dynamics, harmonic free energies and Raman spectra, a discussion on perovskite bond compressibility, and temperature-dependent static structure factors (PDF)
Transparent Peer Review report available (PDF)
