# Correction to “Octahedral Tilt-Driven Phase Transitions in BaZrS_3_ Chalcogenide Perovskite”

**DOI:** 10.1021/acs.jpclett.5c00978

**Published:** 2025-04-23

**Authors:** Prakriti Kayastha, Erik Fransson, Paul Erhart, Lucy Whalley

Correction to *Octahedral Tilt-Driven Phase Transitions inBaZrS*_3_*Chalcogenide*. Published inThe Journal of Physical Chemistry Letters2025, 16( (8), ), 2064–207139971714 10.1021/acs.jpclett.4c03517PMC11873981.

The placements of the inset crystal structures in Figure 1a and Figure 1b are incorrect. They should be displayed as shown in the corrected Figure 1 below.

**Figure 1:**
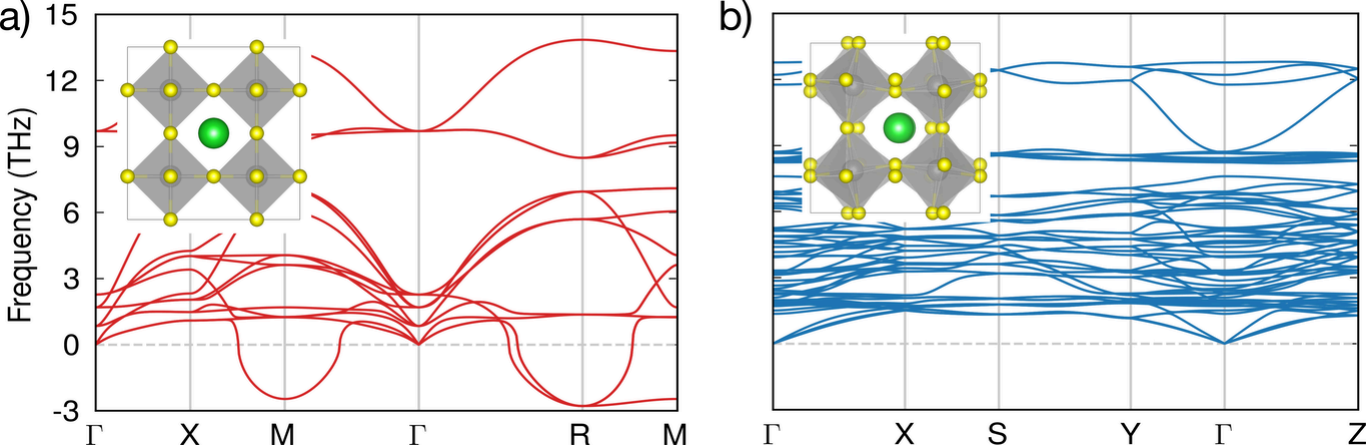
DFT-calculated crystal and phonon band structures of the (a) orthorhombic *Pnma* (b) and cubic *Pm*3̅*m* phases. Green, gray, and yellow spheres represent Ba, Zr, and S atoms, respectively.

In the Discussion we have misspelt an author name; the in-text citation to Reference 32 should read “Jaiswal et al.”.

