# Supplementary material for: Octahedral Tilt-Driven Phase Transitions in BaZrS3 Chalcogenide Perovskite
Source: J Phys Chem Lett. 2025 Feb 19;16(8):2064–71. doi: 10.1021/acs.jpclett.4c03517 (PMC11873981; doi:10.1021/acs.jpclett.4c03517)
Supplement: Supplementary file 1 — jz4c03517_si_001.pdf [file jz4c03517_si_001.pdf]

Supporting Information:

Octahedral tilt-driven phase transitions in $BaZrS_3$ chalcogenide perovskite

Prakriti Kayastha[1], Erik Fransson[2], Paul Erhart[2], and Lucy Whalley[1]

[1] *Department of Physics, Chalmers University of Technology, SE-41296, Gothenburg, Sweden*
[1] *Department of Mathematics, Physics and Electrical Engineering, Northumbria University, Newcastle upon Tyne, NE1 8QH, United Kingdom*

February 7, 2025

Contents

Methods **2**
Density Functional Theory calculations . . . 2
Molecular Dynamics . . . 2

NEP model validation **4**
Comparison of phonon dispersion predicted from DFT and NEP . . . 5

Mode projections for Glazer tilt structures **6**

Recovery of the orthorhombic phase during cooling at $-1\,\mathrm{GPa}$ **7**

Finite-temperature phonons from molecular dynamics **8**

Phase transition temperature using the harmonic approximation **9**

Perovskite bond compressibility **10**

Harmonic Raman spectra **11**

Static structure factor **13**

Partial static structure factors and X-ray diffraction scattering intensities **15**

Supplemental References **17**

Methods

We constructed neuroevolution potential (NEP) models by employing the iterative strategy outlined in Ref. 1. The GPUMD package in version 3.9.4[2–4] was used to build the NEP model and run the molecular dynamics (MD) simulations. The ASE[5] and CALORINE[6] packages were used to prepare the training structures, set up MD simulations and post-process the results.

The initial training set contains strained primitive structures and rattled supercells of the cubic ($Pm\bar{3}m$), tetragonal ($I4/mcm$) and orthorhombic ($Pnma$) perovskite phases, alongside structures corresponding to each of the 15 unique perovskite tilt patterns as specified by Glazer citeglazer1972classification. Random displacements were generated using the HIPHIVE package[7]. The training set also contains 92 Ruddlesden-Popper structures, which will be the subject of a future publication.

The initial model was trained using density functional theory (DFT) data generated with the PBEsol exchange-correlation functional[8]. For each structure in the training set, we calculated the formation energy (relative to the elemental phases), stress tensor and atomic force. This model was then used to run MD simulations in the NPT ensemble, over a temperature and pressure range of 0 K to 1200 K and −5 GPa to 20 GPa respectively, with varying supercell sizes, containing between 20 and 500 atoms. Snapshots from the MD simulations were randomly selected and added to the training set, after which the model was retrained.

Finally, a higher accuracy training set was generated using DFT with the hybrid functional HSE06[9]. Here we carried out single-point calculations on all 1187 training structures generated during the construction of the PBEsol-based model. Stress tensors for structures containing more than 60 atoms were not evaluated as the memory requirements for these calculations were prohibitively large. A comparison of models at the PBEsol level of theory shows that using a sub-set of stress tensors does not impact the predicted phase transition temperature or other properties of interest.

Density Functional Theory calculations

The training data for each NEP model was generated using DFT calculations to evaluate the formation energies (relative to elemental phases), stress tensors and forces. These calculations were performed using the all-electron numeric atom-centered orbital code FHI-aims[10]. FHI-vibes[11] was used for pre and post-processing of DFT data. All DFT calculations used the *light* basis set and a Monkhorst-Pack k-point mesh with a minimum k-spacing of 0.2/Å. For single-point calculations, the charge density was converged to an accuracy of 10^{-6}, forces to 10^{-5} eV/Å and stresses to 10^{-4} eV/Å^3. Geometry relaxations were carried out using the symmetry-constrained relaxation scheme as implemented in ASE[5], until the maximal force component was below 10^{-3} eV/Å.

Harmonic phonon dispersions at 0 K and Helmholtz free energies were evaluated using the PHONOPY package[12] with a $2 \times 2 \times 2$ supercell and a 0.01 Å displacement distance.

Molecular Dynamics

MD simulations were carried out using the GPUMD software[13] with a timestep of 1 fs. The ASE[5] and calorine[6] packages were used to set up the MD simulations and post-process the results. Heating and cooling simulations were run in the NPT ensemble between 0 K to 1200 K for 200 ns using a supercell consisting of about 40 960 atoms. The potential energy and lattice parameters were recorded every 100 fs to discern phase transitions.

Free energy calculations were carried out via thermodynamic integration (TI) using an Einstein crystal as reference Hamiltonian (also referred to as the Frenkel-Ladd method[14]) as outlined in Ref. 15. The free energy of the system described by the NEP, F_{NEP}, is obtained from

$$F_{\mathrm{NEP}} - F_{\mathrm{Ein}} = \int_0^1 \left\langle \frac{\mathrm{d}H(\lambda)}{\mathrm{d}\lambda} \right\rangle_H \mathrm{d}\lambda, \tag{S1}$$

where the integration is carried out over the Kirkwood coupling parameter λ[16], F_{Ein} is the analytically known classical free energy of an Einstein crystal, and the Hamiltonian is $H(\lambda) = (1-\lambda)\, H_{\mathrm{Ein}} + \lambda H_{\mathrm{NEP}}$, as implemented in GPUMD. Here, the ensemble average $\langle \ldots \rangle_H$ is sampled using the Hamiltonian $H(\lambda)$. The Gibbs free energy can then be obtained by $G = F + PV$. TI simulations were

run for 0.05 ns using a spring constant of $4\,\text{eV}/\text{Å}^2$ using a supercell consisting of 23 040 atoms. These simulations were run in the NVT ensemble with lattice parameters obtained from NPT simulations.

The static structure factor, $S(\boldsymbol{q})$, is calculated from NVT simulations using the DYNASOR package[17] as

$$S(\boldsymbol{q}) = \frac{1}{N}\left\langle \sum_i^N \sum_j^N \exp\left[i\boldsymbol{q}\cdot(\boldsymbol{r}_i(t) - \boldsymbol{r}_j(t))\right] \right\rangle, \tag{S2}$$

where $\boldsymbol{r}_i(t)$ is the position of atom i at time t and the sums run over all atoms. To obtain the intensity measured in an X-ray experiment, $I(q)$, one must include the X-ray form factors as

$$I(\boldsymbol{q}) = \frac{1}{N}\left\langle \sum_i^N \sum_j^N f_i(q) f_j(q) \exp\left[i\boldsymbol{q}\cdot(\boldsymbol{r}_i(t) - \boldsymbol{r}_j(t))\right] \right\rangle, \tag{S3}$$

where $f_i(q)$ is the q-dependent X-ray form factors, here taken from Ref. 18. The partial structure factors and intensities can be obtained by considering only specific atom types in the sums, see Ref. 17. Lastly, we apply Bragg's law to convert the structure factor and intensity from q-space to θ-space:

$$\frac{sin(\theta)}{\lambda} = n\frac{q}{4\pi} \tag{S4}$$

where λ is the wavelength of the incident X-ray beam used in experiment.

We employ phonon mode projection to analyze and classify both relaxed structures and snapshots from MD simulations as done in Ref. 19. The atomic displacements $\boldsymbol{u}$ can be projected on a mode λ, with the supercell eigenvector $\boldsymbol{e}_\lambda$, via

$$Q_\lambda = \boldsymbol{u}\cdot\boldsymbol{e}_\lambda$$

Here, the phonon supercell eigenvector of the R and M tilt modes are obtained with PHONOPY[20], and symmetrized such that each of the three degenerate modes corresponds to tilting around the x, y, and z direction respectively.

NEP model validation

Our final training set consists of 1187 structures. Energies, forces, and stress tensors are evaluated using the HSE06 functional. Energies and forces are evaluated for all structures. However, due to large memory requirements, to evaluate stress tensors are only evaluated for structures with less than 60 atoms in the unit cell. Energy and force errors are reported for the entire training set of 1187 structures. Virial and stress errors are only reported for structures where the DFT stress tensors were evaluated.

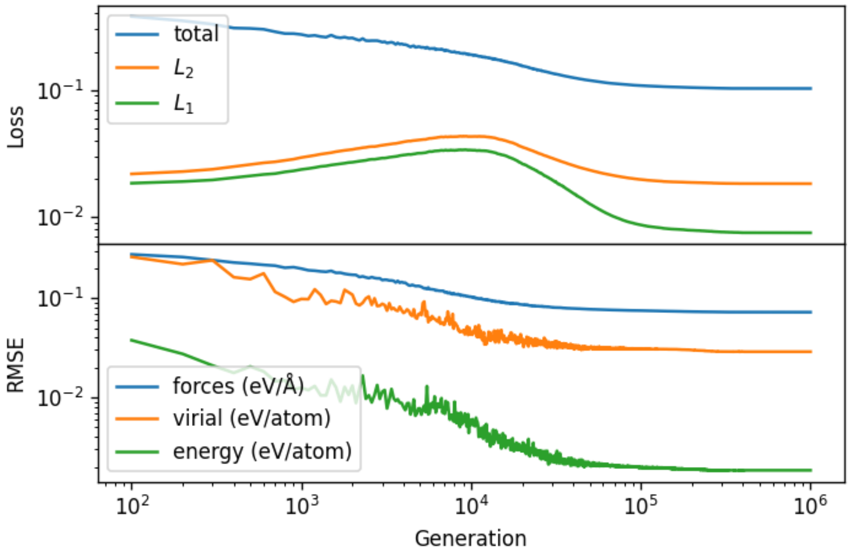

Figure S1: Loss curves for the HSE06 model

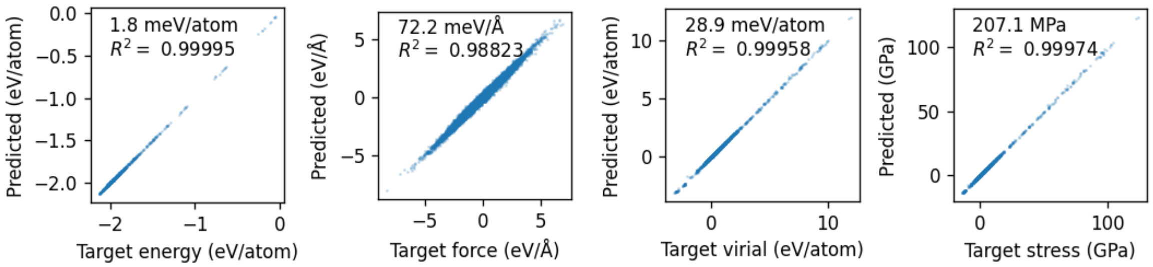

Figure S2: Parity plot for the HSE06 model. Structures for which the stress tensors have not been evaluated with DFT are not included in the virial and stress tensor parity plots.

Comparison of phonon dispersion predicted from DFT and NEP

The 0 K harmonic phonon spectra for $BaZrS_3$ in its three observed phases are displayed. The dashed black line corresponds to that generated with our NEP model, the solid blue is calculated using DFT-calculated forces. The largest discrepancies correspond to high-frequency modes strongly associated with the sulfur species.[21]

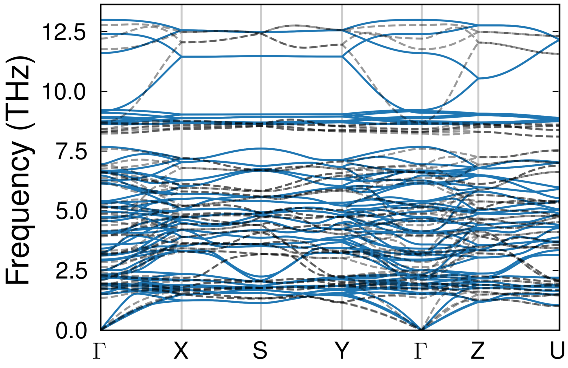

Figure S3: Pnma DFT (solid blue) vs NEP (dashed black) phonons

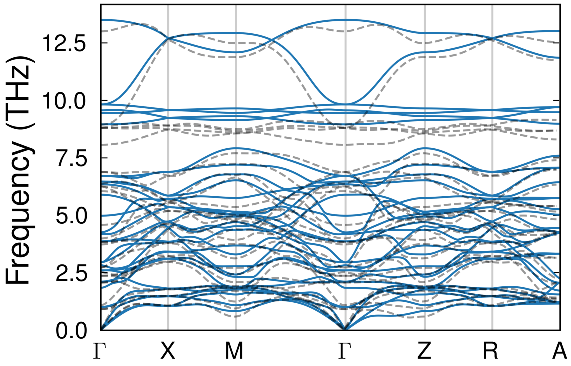

Figure S4: I4/mcm DFT (solid blue) vs NEP (dashed black) phonons

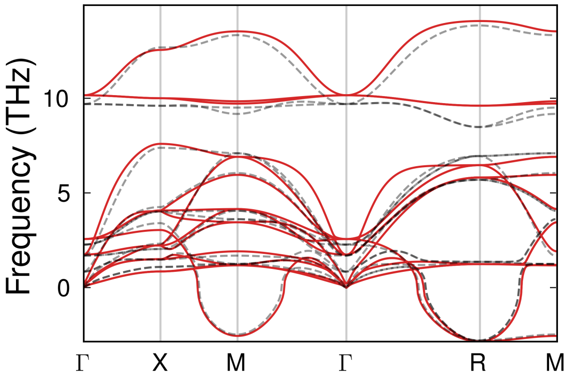

Figure S5: $Pm\bar{3}m$ DFT (solid red) vs NEP (dashed black) phonons

Mode projections for Glazer tilt structures

In our comprehensive evaluation of all 15 Glazer-tilted structures we observe that $I4/mcm$ and its subgroups ($P4_2/nmc$, $Cmcm$, $C2/m$, $C2/c$, $P\bar{1}$) have similar energies according to DFT calculations and the NEP model. Our workflow for constructing these structures was to constrain the spacegroup symmetry using the ASE[5] FixSymmetry function. Subgroup structures which are unstable at 0 K relax to their supergroup phase with a small (but non-zero) phonon mode amplitude. The corresponding distortion lies within the tolerance factor of the symmetry-constrained relaxation (10^{-5} Å). This small difference in structure results in energies that are similar but not exactly the same. For example, the $Cmcm$ $a^0b^+c^-$ structure relaxes to an $I4/mcm$ $a^0b^0c^-$ equivalent structure with a small mode projection value for the b^+ tilt. This results in a small energy difference (0.2 meV) between the two phases.

Similar observations can be made for the $P2_1/m$ and $Pnma$ phases, and the $Immm$ and $P4/mbm$ phases.

Space group	M_x	M_y	M_z	R_x	R_y	R_z
$Pm\bar{3}m$ (221)	0.000000	0.000000	-0.000000	-0.000000	0.000000	0.000000
$I4/mcm$ (140)	0.000000	0.000000	0.000000	0.000000	0.000000	0.575006
$P4/mbm$ (127)	0.000000	0.000000	0.548092	0.000000	0.000000	0.000000
$Imma$ (74)	0.000000	0.000000	0.000000	0.000000	0.373962	0.373962
$C2/m$ (12)	0.000000	0.000000	0.000000	0.000000	0.000030	0.575030
$Cmcm$ (63)	0.000000	0.000002	0.000000	0.000000	0.000000	0.575030
$I4/mmm$ (139)	0.000000	0.328544	0.328544	0.000000	0.000000	0.000000
$R\bar{3}c$ (167)	0.000000	0.000000	0.000000	0.292519	0.292519	0.292519
$C2/c$ (15)	0.000000	0.000000	0.000000	0.575000	0.000016	0.000016
$P\bar{1}$ (2)	0.000000	0.000000	0.000000	0.574996	0.000037	0.000017
$I4/mmm$ (139)	0.000000	0.328544	0.328544	0.000000	0.000000	0.000000
$Pnma$ (62)	0.401595	0.000000	0.000000	0.000000	0.321830	0.321830
$P2_1/m$ (11)	0.401610	0.000000	0.000000	0.000000	0.321820	0.321830
$P4_2/nmc$ (137)	0.000001	0.000001	0.000000	0.000000	0.000000	0.575031
$Im\bar{3}$ (204)	0.251797	0.251797	0.251797	0.000000	0.000000	0.000000
$Immm$ (71)	0.548063	0.000001	0.000003	0.000000	0.000000	0.000000

Table S1: Mode projection on DFT relaxed geometries

Space group	M_x	M_y	M_z	R_x	R_y	R_z
$Pm\bar{3}m$ (221)	0.000000	0.000000	0.000000	0.000000	0.000000	0.000000
$I4/mcm$ (140)	0.000000	0.000000	0.000000	0.000000	0.000000	0.579234
$P4/mbm$ (127)	0.000000	0.000000	0.542603	0.000000	0.000000	0.000000
$Imma$ (74)	0.000000	0.000000	0.000000	0.000000	0.369080	0.369080
$C2/m$ (12)	0.000000	0.000000	0.000000	0.000000	-0.000028	0.579234
$Cmcm$ (63)	0.000000	0.000000	0.000000	0.000000	0.000000	0.579234
$I4/mmm$ (139)	0.000000	0.329391	0.329391	0.000000	0.000000	0.000000
$R\bar{3}c$ (167)	0.000000	0.000000	0.000000	0.286208	0.286208	0.286208
$C2/c$ (15)	0.000000	0.000000	0.000000	0.579234	0.000025	0.000025
$P\bar{1}$ (2)	0.000000	0.000000	0.000000	0.579233	0.000045	0.000011
$I4/mmm$ (139)	0.000000	0.329391	0.329391	0.000000	0.000000	0.000000
$Pnma$ (62)	0.400823	0.000000	0.000000	0.000000	0.326056	0.326056
$P2_1/m$ (11)	0.400821	0.000000	0.000000	0.000000	0.326061	0.326054
$P4_2/nmc$ (137)	0.000000	0.000000	0.000000	0.000000	0.000000	0.579234
$Im\bar{3}$ (204)	0.251378	0.251378	0.251378	0.000000	0.000000	0.000000
$Immm$ (71)	0.542603	0.000000	0.000000	0.000000	0.000000	0.000000

Table S2: Mode projection on NEP relaxed geometries

Recovery of the orthorhombic phase during cooling at $-1\,\mathrm{GPa}$

Our cooling simulations in the main text do not recover the orthorhombic Pnma phase formed at 0 K. This stems from the numerical limitations of our simulations; the short timescales considered make a first-order transition (with associated kinetic barrier) from the tetragonal phase implausible. Here, we plot the heating and cooling runs at 1 GPa, where the orthorhombic phase is recovered around 560 K. This variation in our results is due to the stochastic nature of molecular dynamics simulations.

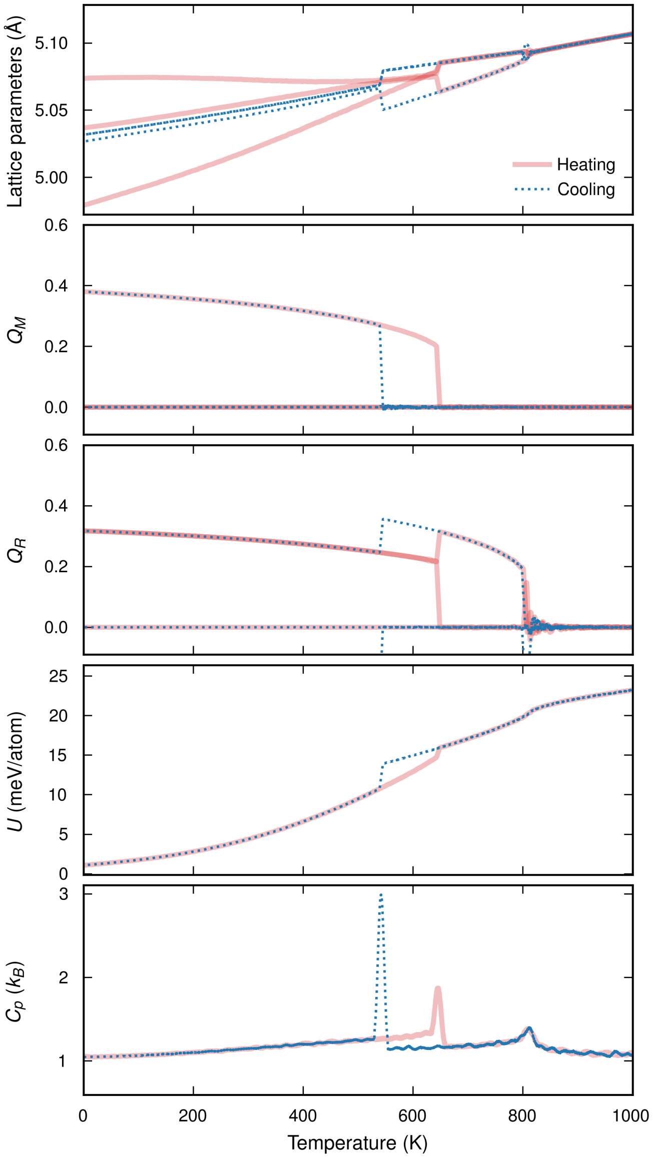

Figure S6: Heating and cooling runs at pressure $-1\,\mathrm{GPa}$. The orthorhombic Pnma phase is recovered in the cooling run, unlike our simulations at 0 Pa (see the main text).

Finite-temperature phonons from molecular dynamics

The DYNASOR package[17] is used to calculate the spectral energy density of the $I4/mcm$ and $Pm3m$ phases at various temperatures.

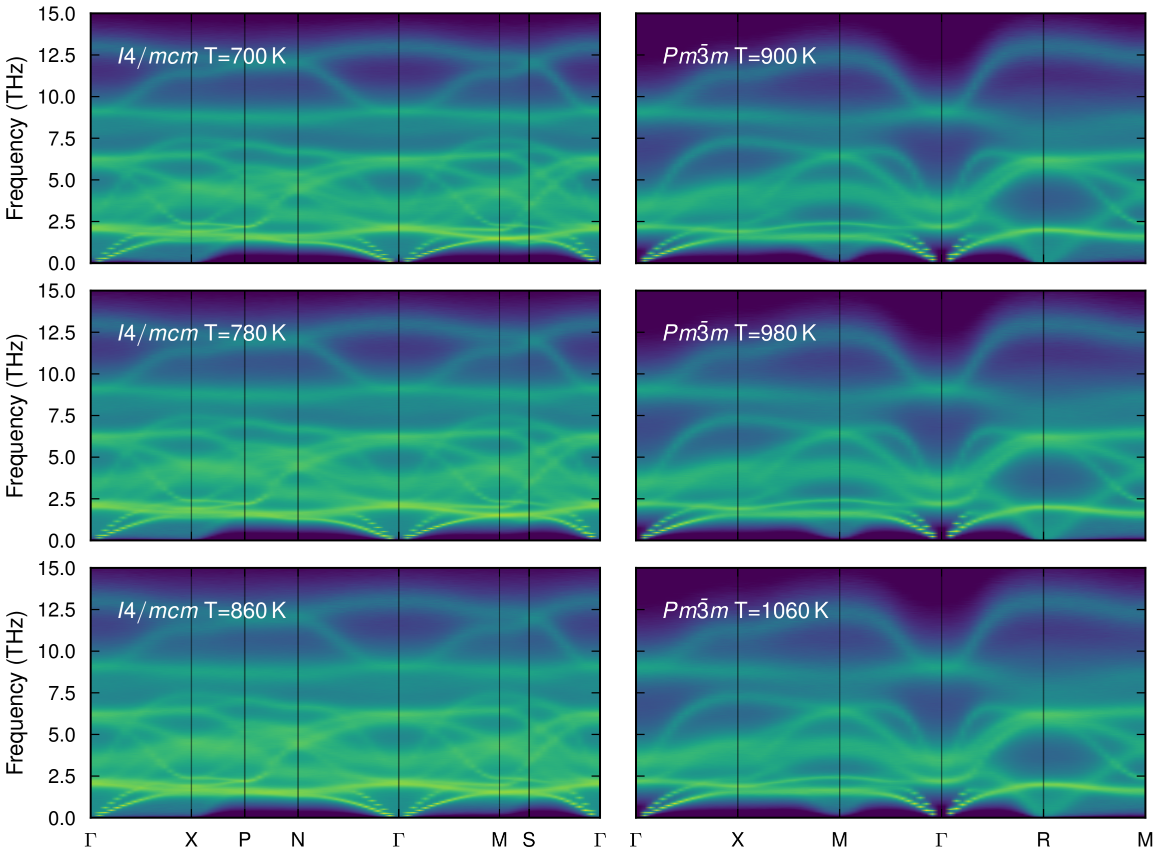

Figure S7: Spectral energy densities of the tetragonal $I4/mcm$ and cubic $Pm\bar{3}m$ phases of $BaZrS_3$.

Phase transition temperature using the harmonic approximation

Helmholtz free energies of the orthorhombic ($Pnma$) and tetragonal ($I4/mcm$) phases are evaluated within the harmonic approximation. The phase transition temperature with DFT-calculated phonon frequencies is 460 K, and with NEP-calculated frequencies it is 243 K. The phase transition temperature predicted with our fully anharmonic molecular dynamics model (see main text) is 610 K.

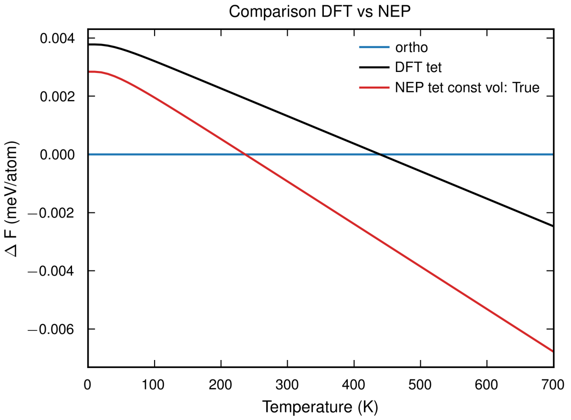

Figure S8: Helmholtz free energy difference between $Pnma$ and $I4/mcm$ phases (ΔF), calculated under the harmonic approximation.

Perovskite bond compressibility

Perovskites with lower symmetry (more tilted) structures generally exhibit a series of octahedral tilt-driven phase transitions to higher symmetry (less tilted) structures with increasing temperature. The phase transition temperature T_c can increase or decrease with pressure, $\frac{dT_c}{dP} > 0$ or $\frac{dT_c}{dP} < 0$ respectively.[22] For $BaZrS_3$ we find $\frac{dT_c}{dP} > 0$ (Fig 3 in the main text). This behaviour can be rationalised by considering the relative compressibility of the A-X and B-X bonds.[22,23] If the B-X bonds forming the octahedra are relatively rigid then pressure must induce tilting to accommodate the volume reduction, resulting in the compression of the A-X bonds and a reduction in symmetry. If the A-X bonds are relatively rigid then pressure must induce a reduction in the octahedral volume through compression of the B-X bond. The ratio of the B-X and A-X bond compressibilities ($\frac{\beta_B}{\beta_A}$) can be used to indicate how the phase transition temperature will vary as a function of pressure: $\frac{dT_c}{dP} > 0$ when $\frac{\beta_B}{\beta_A} < 1$, and $\frac{dT_c}{dP} < 0$ when $\frac{\beta_B}{\beta_A} > 1$. The parameter $M_i = \beta_i^{-1}$ is given by:

$$M_i = \frac{R_i N_i}{B} \exp\left(\frac{R_i - R_0}{B}\right) \tag{S5}$$

where N_i is the coordination number, R_i average bond distance (to X-atoms), B is a constant with value 0.37, and R_0 is the bond-valence parameter.[24] For $BaZrS_3$ we obtain $\frac{\beta_B}{\beta_A} = \frac{M_A}{M_B} \approx 0.45$. This indicates that $\frac{dT_c}{dP} > 0$, in agreement with our predicted phase diagram.

Harmonic Raman spectra

The following harmonic Raman spectra analysis was performed using the phonopy-spectroscopy code.[25] Peak positions were evaluated assuming harmonic vibrational behaviour and using the PBEsol functional. Dielectric tensors were evaluated using the PBE functional. A light basis set with a minimum density of 5 k-grids per $Å^{-1}$ was used throughout. A Lorentzian of width $0.05 cm^{-1}$ is used to broaden the peaks.

$BaZrS_3$ in the the *Pnma* phase has 60 optic phonon modes, of which 24 are Raman active: $\Gamma = 7A_g \bigoplus 5B_{1g} \bigoplus 7B_{2g} \bigoplus 5B_{3g}$.

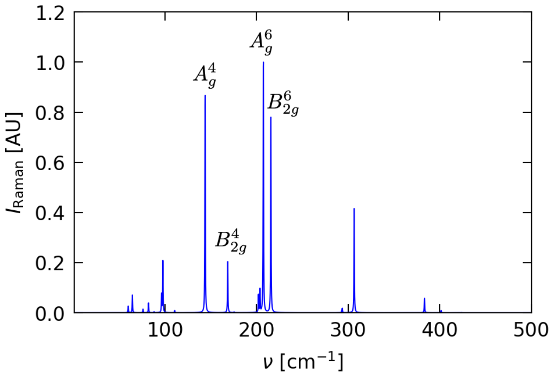

Figure S9: Harmonic first-order Raman spectra of the *Pnma* phase

Frequency [cm^{-1}]	Intensity [$Å^4$ amu^{-1}]	Mode Symmetry
57.38246	31.294167	A_g
62.740697	74.397711	B_{2g}
72.865691	20.712113	A_g
74.878717	0.428877	B_{3g}
79.451252	35.213175	B_{2g}
82.730798	4.047662	B_{1g}
89.396457	81.906317	B_{2g}
94.004782	202.648574	A_g
105.117071	8.151943	B_{1g}
139.776095	927.058612	A_g
157.205497	0.129784	B_{3g}
162.91409	192.607928	B_{2g}
166.327512	3.336429	A_g
171.34857	2.338391	B_{1g}
198.430549	98.18659	B_{2g}
200.803735	93.792521	B_{3g}
204.900935	1063.364972	A_g
213.109461	766.788035	B_{2g}
289.681849	19.314493	B_{1g}
289.943548	8.201392	B_{3g}
304.087743	361.586611	A_g
389.08038	48.066782	B_{1g}
400.954212	7.889141	B_{2g}
419.617256	0.634279	B_{3g}

Table S3: Raman frequencies and mode symmetries for the *Pnma* phase

$BaZrS_3$ in the $I4/mcm$ phase has 30 optic phonon modes, of which 7 are Raman active: $\Gamma = 3E_g \bigoplus A_{1g} \bigoplus 2A_{2g} \bigoplus B_{1g} \bigoplus B_{2g}$.

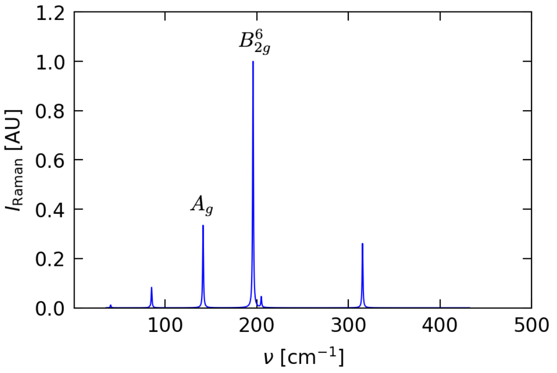

Figure S10: Harmonic first-order Raman spectra of the $I4/mcm$ phase

Frequency [cm^{-1}]	Intensity [$Å^4$ amu^{-1}]	Mode Symmetry
40.762563	3.683262	E_g
40.762563	3.687507	E_g
42.565308	0.000000	A_{2u}
69.245268	0.000000	E_u
69.245268	0.000000	E_u
83.275265	0.896384	E_g
83.275265	0.894630	E_g
85.401421	53.217047	B_{2g}
100.908270	0.000000	E_u
100.908270	0.000000	E_u
126.898251	0.000000	E_u
126.898251	0.000000	E_u
137.013961	0.000000	A_{2u}
141.559757	216.862006	A_{1g}
155.032805	0.000000	B_{1u}
196.069483	645.987936	B_{2g}
205.046737	13.904421	E_g
205.046737	13.919185	E_g
207.325511	0.000000	A_{1u}
230.492719	0.000000	E_u
230.492719	0.000000	E_u
281.507146	0.000044	A_{2g}
302.441159	0.000000	A_{2u}
303.618329	0.000000	E_u
303.618329	0.000000	E_u
315.495626	167.934156	B_{1g}
427.229088	0.000392	A_{2g}

Table S4: Raman frequencies and mode symmetries for the $I4/mcm$ phase

$BaZrS_3$ in the $Pm\bar{3}m$ phase has 15 optic phonon modes however, due to Raman selection rules, none of the modes are first-order Raman active. Any Raman signal observed from this phase derive from second-order Raman effects.

Static structure factor

X-ray scattering intensities are equivalent to static structure factors weighted with species- and source-dependent form factors. We demonstrate the effect of including the form factors through direct comparison in Fig. S11. A number of peaks are not present in the scattering intensity, most noticeably at a scattering angle of 17.5°.

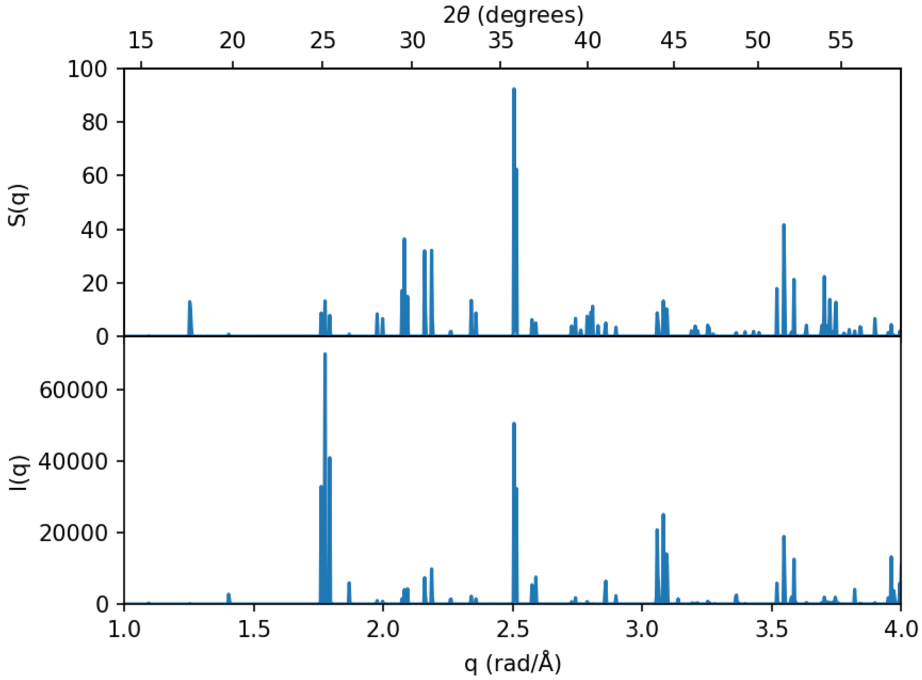

Figure S11: Comparison of the static structure factor pattern (S(q)) to the x-ray diffraction pattern (I(q)).

The temperature-dependent static structure factors are displayed in Fig. S12. In contrast to the main text, superlattice peaks up to the fifth Brillouin Zone are displayed.

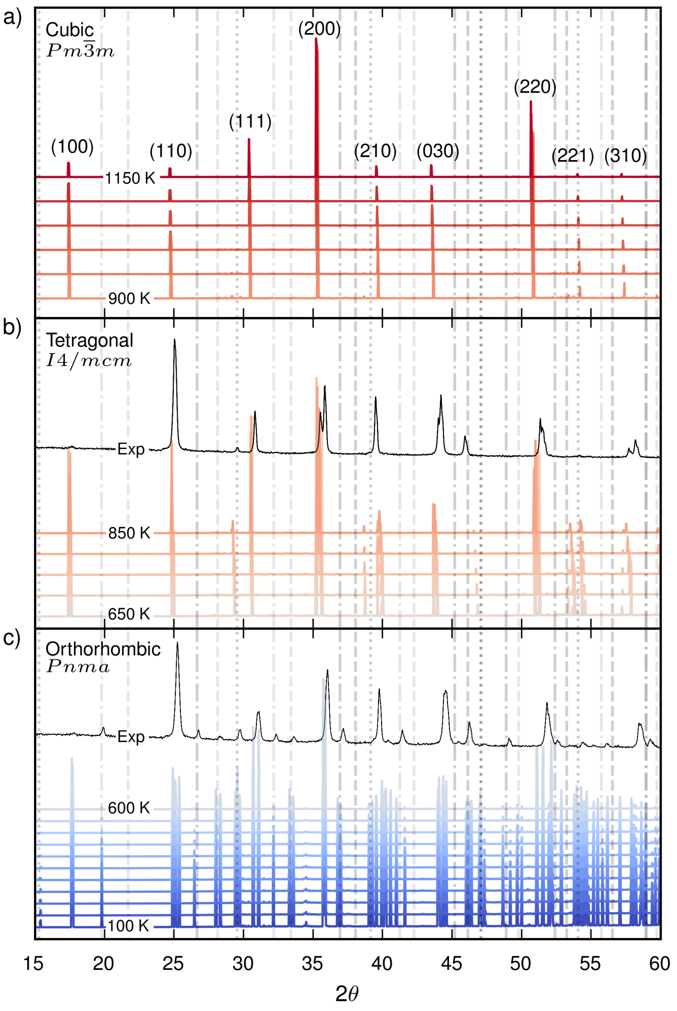

Figure S12: Static structure factor, $S(q)$, evaluated for three $BaZrS_3$ polymorphs from 100 K to 1150 K in intervals of 50 K. A Cu Kα value of 1.5406 Å was used for the q to θ conversion. Cubic $Pm\bar{3}m$ peaks are indexed. Superlattice peaks (up to the fifth Brillouin Zone) associated with R-, M- and X-point distortions are indexed and marked with dotted, dashed, and dash-dotted lines, respectively. Experimental XRD data of the orthorhombic (303 K) and tetragonal (923 K) phases from Ref. 26 are plotted in black.

Partial static structure factors and X-ray diffraction scattering intensities

We can decompose the static structure factors for the various atom types in the system to compute partial static structure factors. In Fig. S13 we plot the partial structure factor of the $Pnma$ phase at 300 K and 0 Pa. Similarly, we can decompose the X-ray diffraction scattering intensity $I(q)$. This is displayed in Fig. S14.

In Fig. S13 and Fig. S14 there is a peak at $q = 1.4$ for the Ba-Ba partials. This corresponds to a distortion at the X-point which involves atomic displacement of the Ba species only.

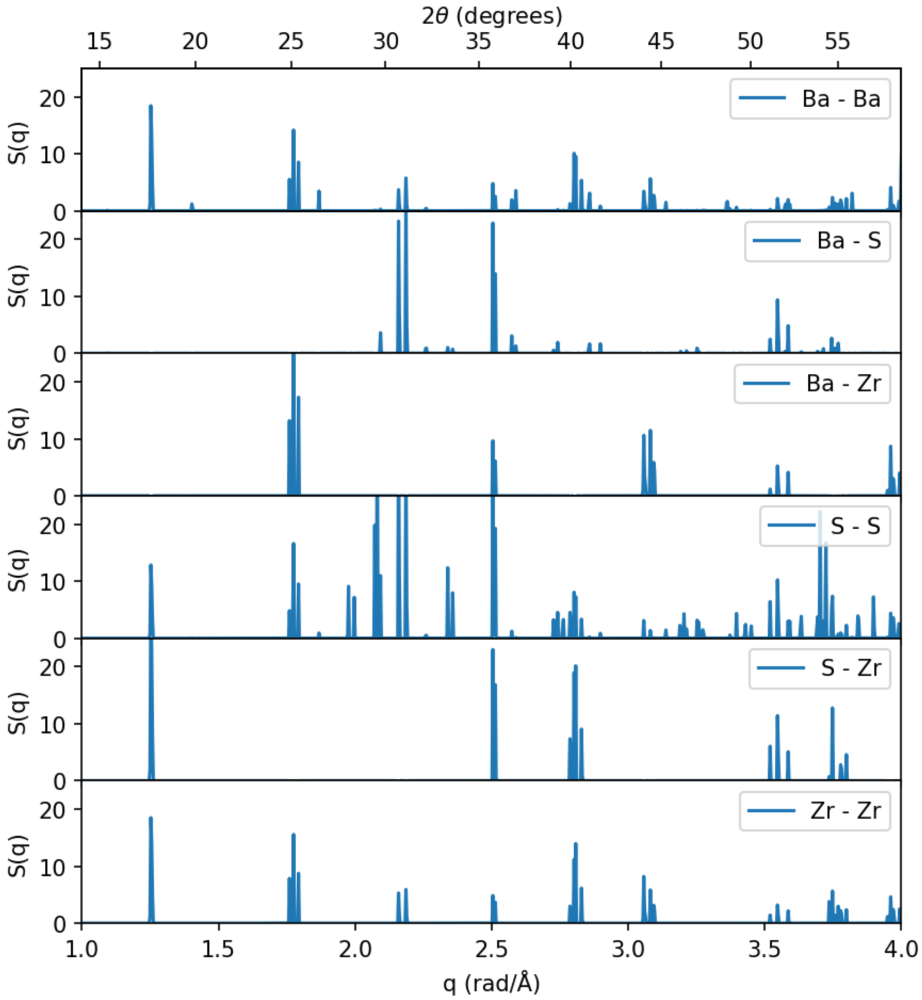

Figure S13: Partial static structure factors, $S(q)$, for $BaZrS_3$ in the $Pnma$ phase at 300 K and 0 Pa.

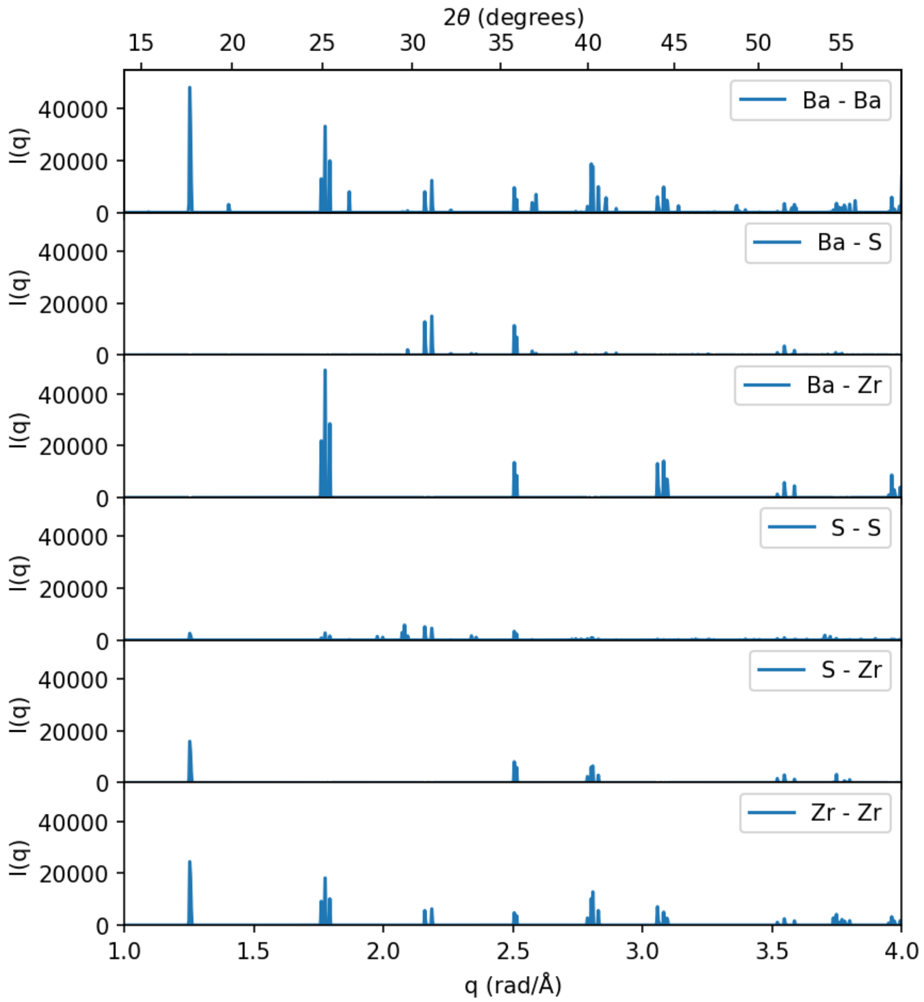

Figure S14: Partial scattering intensities, $I(q)$, for $BaZrS_3$ in the $Pnma$ phase at 300 K and 0 Pa.

Supplemental References

[1] E. Fransson, J. Wiktor, and P. Erhart, Phase transitions in inorganic halide perovskites from machine learning potentials (2023), arXiv.2301.03497.

[2] Z. Fan, Z. Zeng, C. Zhang, Y. Wang, K. Song, H. Dong, Y. Chen, and T. Ala-Nissila, Physical Review B **104**, 104309 (2021).

[3] Z. Fan, Journal of Physics: Condensed Matter **34**, 125902 (2022).

[4] Z. Fan, Y. Wang, P. Ying, K. Song, J. Wang, Y. Wang, Z. Zeng, K. Xu, E. Lindgren, J. M. Rahm, A. J. Gabourie, J. Liu, H. Dong, J. Wu, Y. Chen, Z. Zhong, J. Sun, P. Erhart, Y. Su, and T. Ala-Nissila, The Journal of Chemical Physics **157**, 114801 (2022).

[5] A. H. Larsen, J. J. Mortensen, J. Blomqvist, I. E. Castelli, R. Christensen, M. Dułak, J. Friis, M. N. Groves, B. Hammer, C. Hargus, E. D. Hermes, P. C. Jennings, P. B. Jensen, J. Kermode, J. R. Kitchin, E. L. Kolsbjerg, J. Kubal, K. Kaasbjerg, S. Lysgaard, J. B. Maronsson, T. Maxson, T. Olsen, L. Pastewka, A. Peterson, C. Rostgaard, J. Schiøtz, O. Schütt, M. Strange, K. S. Thygesen, T. Vegge, L. Vilhelmsen, M. Walter, Z. Zeng, and K. W. Jacobsen, Journal of Physics: Condensed Matter **29**, 273002 (2017).

[6] E. Lindgren, M. Rahm, E. Fransson, F. Eriksson, N. Österbacka, Z. Fan, and P. Erhart, Journal of Open Source Software **9**, 6264 (2024).

[7] F. Eriksson, E. Fransson, and P. Erhart, Advanced Theory and Simulations **2**, 1800184 (2019).

[8] J. P. Perdew, A. Ruzsinszky, G. I. Csonka, O. A. Vydrov, G. E. Scuseria, L. A. Constantin, X. Zhou, and K. Burke, Physical review letters **100**, 136406 (2008).

[9] A. V. Krukau, O. A. Vydrov, A. F. Izmaylov, and G. E. Scuseria, J. Chem. Phys. **125**, 224106 (2006).

[10] V. Blum, R. Gehrke, F. Hanke, P. Havu, V. Havu, X. Ren, K. Reuter, and M. Scheffler, Computer Physics Communications **180**, 2175 (2009).

[11] F. Knoop, T. Purcell, M. Scheffler, and C. Carbogno, The Journal of Open Source Software **5** (2020).

[12] A. Togo, Journal of the Physical Society of Japan **92**, 012001 (2023).

[13] Z. Fan, W. Chen, V. Vierimaa, and A. Harju, Computer Physics Communications **218**, 10 (2017).

[14] D. Frenkel and A. J. C. Ladd, The Journal of Chemical Physics **81**, 3188 (1984).

[15] R. Freitas, M. Asta, and M. de Koning, Computational Materials Science **112**, 333 (2016).

[16] J. G. Kirkwood, The Journal of Chemical Physics **3**, 300 (1935).

[17] E. Fransson, M. Slabanja, P. Erhart, and G. Wahnström, Advanced Theory and Simulations **4**, 2000240 (2021).

[18] D. Waasmaier and A. Kirfel, Acta Crystallographica Section A Foundations of Crystallography **51**, 416–431 (1995).

[19] E. Fransson, P. Rosander, F. Eriksson, J. M. Rahm, T. Tadano, and P. Erhart, Communications Physics **6**, 173 (2023).

[20] A. Togo and I. Tanaka, Scripta Materialia **108**, 1 (2015).

[21] Y. Wu, Y. Chen, Z. Fang, Y. Ding, Q. Li, K. Xue, H. Shao, H. Zhang, and L. Zhou, The Journal of Physical Chemistry Letters **14**, 11465 (2023).

[22] R. J. Angel, J. Zhao, and N. L. Ross, Physical review letters **95**, 025503 (2005).

[23] J. Zhao, N. L. Ross, and R. J. Angel, Journal of Physics: Condensed Matter **16**, 8763 (2004).

[24] I. D. Brown and D. Altermatt, Acta Crystallographica Section B **41**, 244 (1985).

[25] J. M. Skelton, L. A. Burton, A. J. Jackson, F. Oba, S. C. Parker, and A. Walsh, Physical Chemistry Chemical Physics **19**, 12452 (2017).

[26] R. Bystrický, S. K. Tiwari, P. Hutár, and M. Sýkora, Inorganic Chemistry **63**, 12826 (2024).
