# Supplementary material for: Octahedral Tilt-Driven Phase Transitions in BaZrS3 Chalcogenide Perovskite
Source: J Phys Chem Lett. 2025 Feb 19;16(8):2064–71. doi: 10.1021/acs.jpclett.4c03517 (PMC11873981; doi:10.1021/acs.jpclett.4c03517)
Supplement: Supplementary file 2 — jz4c03517_si_002.pdf [file jz4c03517_si_002.pdf]

jz-2024-03517f.R1

Name: Peer Review Information for "Octahedral tilt-driven phase transitions in BaZrS₃ chalcogenide perovskite"

First Round of Reviewer Comments

Reviewer: 1

Comments to the Author

The chalcogenide perovskite BaZrS3 is a novel semiconductor which has recently attracted considerable attention for photovoltaic and thermoelectric applications. Synthesizing high-quality BaZrS3 samples remains challenging as it requires high temperatures. This highlights the need for clarifying possible polymorphic phase transitions and the phase diagram of BaZrS3. In this work, the authors theoretically provide this timely understanding based on MD simulations and group-subgroup analysis. In particular, the authors develop a machine learning interatomic potential to drive the large-scale MD simulations. This theoretical research is sound, and the writing is easy to follow. The method used in this work would be interesting for broad readers, considering the polymorphism popular in many important functional materials (such as hybrid perovskites, inorganic solar cell absorber Cu2ZnSnS4, and wide-band-gap oxide Ga2O3). I recommend publishing this work in The Journal of Physical Chemistry Letters after addressing the below suggested minor revisions and questions.

1. “An understanding of the exact BaZrS3 perovskite structure is important as a phase transition within the operational temperature range of solar cells (−20 ◦C to 60 ◦C) or thermoelectric generators (100 ◦C to 800 ◦C) might impact key functional properties”. It seems unlikely that BaZrS3 would undergo phase transitions under typical solar cell operating conditions. Rather, should it be more concerned about obtaining phase-pure BaZrS3 samples? given the high-temperature growth.

2. Do the findings imply that if BaZrS3 is grown under high temperature, the resulting sample could be a mixture of different polymorphs? In addition, which are more worrisome, the polymorphic phases or Ruddlesden–Popper phases?

3. I noticed a very recent experimental work (High-Temperature Polymorphism and Band-Gap Evolution in BaZrS3, https://pubs.acs.org/doi/full/10.1021/acs.inorgchem.4c03895). This work,

based on the experimental techniques as discussed by the authors, observed a Cmcm phase of $BaZrS_3$. I suggest the authors briefly discuss this experimental work.

4. Given the phonon calculations at hand, the authors could add a table in the SI pointing out the Raman active modes for the different polymorphic phases of $BaZrS_3$, which will help future experimental characterizations. This could be important, given there are still theoretical and experimental gaps.

5. I suggest the authors restructure the first paragraph of the Methods section to be four parts: (i) a one to two sentence short introduction, (ii) the 1187 atomic structures and DFT calculations, (iii) the development of the ML interatomic potential and its accuracy, and (iv) MD settings and simulations (NPT, NVT). While details could be found in the SI, it is still important to make clear for each part the purpose and the quantities computed. In addition, I think the tables and figures in the SI should be mentioned at least once in the main text.

6. While the writing is clear, it could still be overall polished for better readability and higher quality. For example, in the abstract, "Phase transitions of the $BaZrS_3$ perovskite are under-explored in literature as most experimental characterization is performed at ambient conditions" could be "Phase transitions of $BaZrS_3$ remain underexplored in literature, as most experimental characterizations of this material have been performed at ambient conditions". This is just one example and my suggested modifications (and I believe the authors would have better modifications). Besides, I think there are some typos, in Figure 1, the Glazer notion of the Pnma phase is $a^+b^-b^-$, while in the main text, it is $a^-a^-c^+$. Additionally, (i) I think the citation numbers in a single sentence should generally be put together, (ii) It would be better to consistently put "interatomic" before "potential", (iii) it would be more precise to say "free energies", "atomic forces", and "stress tensors", and (iv) It is not necessary to add an "NEP" before "machine learning".

7. "In Fig. 2, we plot properties observed and derived from MD simulations spanning 0 K to 1200 K and 200 ns." If NPT MD is performed here, it is necessary to make clear the pressure.

8. "All quantities are averaged over a time period corresponding to 5 K." What does "corresponding to 5 K" mean?

9. "This might contribute towards the quantitative discrepancy between the experimentally measured transition at 773 K, 30 and our predicted transition at 610 K." I guess this is a discussion of the triangle data point in Figure 3, so it is necessary to refer to Figure 3 here.

10. “Both phase transitions occur above 600 K, which agrees with experimental characterisation showing BaZrS3 forms in the orthorhombic Pnma phase at ambient temperature and pressure.” It seems more appropriate to use “is stable” or “exists” instead of “forms”.

Reviewer: 2

Comments to the Author

Review of jz-2024-03517f

The manuscript presents a comprehensive study on the structure and properties of the chalcogenide perovskite $BaZrS_3$ under varying temperature and pressure conditions, employing machine learning models trained on MD and DFT data.

The study reveals that $BaZrS_3$ undergoes a first-order orthorhombic-to-tetragonal phase transition at 650 K and a second-order tetragonal-to-cubic phase transition at 850 K. These transitions are evidenced by changes in lattice parameters, heat capacity, mode amplitudes, and structure factor.

Additionally, the authors identified the space groups associated with each stable phase and constructed a temperature-pressure phase diagram for $BaZrS_3$. The findings are further validated through comparison with experimental observations.

I recommend this manuscript for publication, provided the authors address the specific issues and questions highlighted in the review.

General Comment:

Admittedly, I am an experimentalist and, therefore, cannot review the quality or validity of the calculations. However, I can judge the manuscript as a scientist who would want to read and learn from it.
The manuscript presents important and interesting results, addressing a relevant problem using a relatively new calculation.

It is well-written and argued, the figures are generally readable, and the authors have done a great job of relating their findings to experimental results.

Specific Comments:

- **Calorimetric Measurements:** The authors have calculated the heat capacity of $BaZrS_3$ but did not refer to experimental work on calorimetric measurements such as Ref. 56. Moreover, the authors in Ref. 56 have attributed the features in the TGA and DSC of $BaZrS_3$ as precursors leaving the sample and oxidation and not a phase transition.
- **Discussion on Figure 2:**
  - The authors claim to show the activation of modes in Figure 2 b,c. The concept of mode activation usually refers to selection rules of specific measurements. It is not clear what the authors mean by 'active'.
  - In the discussion on Figure 2 b,c, the authors should give references or additional explanations to the claims that mode activation correlates to the existence of specific phases.
- **Hysteresis of the First-order Transition:** The authors should reference/explain the stochastic nature of MD simulations and why they result in hysteresis in their cooling runs.
- **Typo:** On page 5, at the end of the left column, there's a typo - "see Sect. for further discussion" – the section number is missing.
- **First-order transition in Raman:** It is widely acceptable that the Raman signature of tetragonal and cubic phases in perovskites are broad and very di[erent than the orthorhombic phase. This is due to the dynamic stabilization required related to the imaginary modes. Therefore, to claim that the tetragonal and orthorhombic phases of $BaZrS_3$ would have a similar Raman, I expect to see a calculation with no imaginary modes or at least the Gamma point mode frequencies of one.
- **Negative pressures?** A general question as an experimentalist regarding the calculation and Figure 3 specifically – What's the meaning of negative pressures? Why not focus the calculations on feasible pressures that one may measure or work at? Perhaps add a discussion on the significance of phase transitions at high/negative pressures on the functionality and properties of the material.

Author's Response to Peer Review Comments:

Dear Editor and team,

Please find our response attached.

Best Regards,

Dr Lucy Whalley (on behalf of all authors)

Northumbria University

Newcastle upon Tyne

NE1 8ST

l.whalley@northumbria.ac.
uk https://lucydot.github.io

February 7, 2025

Dr Lucy Whalley PhD MInstP MRSC

Assistant Professor in Physics

Dear Editor,

We have uploaded the revised manuscript "**Octahedral tilt-driven phase transitions in $BaZrS_3$ chalcogenide perovskite**". We have very much taken on board the reviewer suggestions – our detailed response is below.

We would like to thank your office and the reviewers for taking the time to consider our manuscript, and hope you consider the work suitable for The Journal of Physical Chemistry Letters.

Best regards,

Dr Lucy Whalley (on behalf of all authors)

<u>Detailed Response</u>

We thank the reviewers for their positive comments and their detailed and expert consideration of this work, which has enabled us to make a marked improvement to the manuscript.

In addition to the changes made in response to our reviewers we have improved our analysis displayed in Figure 4. Rather than showing the static structure factor, we now display the X-ray scattering intensity. This takes account of the atomic form factors for each species, allowing for a

more direct comparison to published experimental results. The static structure factor has been moved to the Supplementary Information.

All changes are highlighted in the attached pdf.

Reviewer 1

1) *"An understanding of the exact $BaZrS_3$ perovskite structure is important as a phase transition within the operational temperature range of solar cells (−20 ◦C to 60 ◦C) or thermoelectric generators (100 ◦C to 800 ◦C) might impact key functional properties". It seems unlikely that $BaZrS_3$ would undergo phase transitions under typical solar cell operating conditions. Rather, should it be more concerned about obtaining phase-pure $BaZrS_3$ samples? given the high-temperature growth. Do the findings imply that if $BaZrS_3$ is grown under high temperature, the resulting sample could be a mixture of different polymorphs?*

Thank you for raising this question. Our simulations do not give insight into this, as the hysteresis effect on cooling stems from the numerical limitations associated with molecular dynamics simulations. We note there is some literature relating to this, which we now cite, and we now suggest it as an area for future study.

We have updated the wording in the introductory section as follows (along with additional references as specified in the manuscript): "Phase transitions may occur before reaching the elevated temperatures required for $BaZrS_3$ synthesis (>850 K). In this case it is possible that samples grown at high-T may include mixtures of polymorphs, as has been observed for halide perovskites. A phase transition within the operating temperature range for thermoelectric generators (400 K to 1100 K) is also possible. An understanding of the exact $BaZrS_3$ perovskite structure is important as even small changes to structure can impact key functional properties including the band gap.

In the conclusion we have added: "It is possible that a $BaZrS_3$ sample grown at high temperature may include mixtures of polymorphs. Future work might more fully consider polymorph mixing, alongside the impact of octahedral tilting on the thermal and optoelectronic properties of $BaZrS_3$.

2) *In addition, which are more worrisome, the polymorphic phases or Ruddlesden–Popper phases?*

In the conclusion we have added (along with additional references as specified in the manuscript): "We note that the formation of ternary Ruddlesden-Popper phases have been considered elsewhere in the literature. When formed these are likely to have greater impact on material properties through disruption of the 3D octahedral framework."

3) *I noticed a very recent experimental work (High-Temperature Polymorphism and Band-Gap Evolution in $BaZrS_3$). This work, based on the experimental techniques as discussed by the authors, observed a Cmcm phase of $BaZrS_3$. I suggest the authors briefly discuss this experimental work.*

Thank you for highlighting this recent journal article from Jaiswal et al.

Given its high relevance to our work, we have included an additional paragraph in our Discussion: "A multimodal study combining synchrotron XRD, Raman spectroscopy, optical measurements and thermal analysis as a function of the temperature identifies three polymorphs when BaZrS3 is heated in air. Rietveld analysis of the synchrotron powder X-ray diffraction patterns shows that the *I4/mcm* phase is stable above 770 K and that the *Pnma* phase is stable below 570 K. From 570 K to 770 K indirect observations suggest that *Cmcm* co-exists as a minority phase. Despite including the *Cmcm* phase in our training data, our simulations do not predict *Cmcm* as a stable intermediate phase. In fact, at 0 K our DFT calculations show that this phase is kinetically unstable and relaxes to form the higher symmetry *I4/mcm* phase (Table S1)."

We have also included a short discussion on another highly relevant paper published after our submission (https://www.nature.com/articles/s43246-024-00705-y): "A recent computational study predicts that the polar *P na21* phase is 0.05 meV per formula unit more stable than *Pnma* at 0 K. This instability has been observed across a variety of oxide perovskites in the orthorhombic phase. For BaZrS3 the small 0.05 meV energy difference follows small differences in atomic co-ordinates, with an extremely tight symmetry tolerance of 0.003 Angstrom required to differentiate between each phase."

4) *Given the phonon calculations at hand, the authors could add a table in the SI pointing out the Raman active modes for the different polymorphic phases of BaZrS3, which will help future experimental characterizations. This could be important, given there are still theoretical and experimental gaps.*

In response to your suggestion, and the newly published paper from Jaiswal et al highlighted in the previous point, we have extended our analysis of the Raman response. This includes identifying the Raman active modes for the *Pnma* and *I4/mcm* phases, calculating the harmonic Raman response, and comparing our predictions against experiment.

To present these results we have extended a paragraph in the main text and included two additional figures and tables in the SI: "Whilst Ye et al. report that there is no indication of a first-order phase transition between 10 K to 875 K, Jaiswal et al. find that the number of Raman peaks decreases with increasing temperature, indicative of a phase transition to a higher-symmetry structure. Our simulated Raman spectra in Fig. S9 and Fig. S10 demonstrates that there is significant peak overlap between the *Pnma* and I*4/mcm* phases. Our spectra also reproduces the two most pronounced changes with temperature from Jaiswel et al.: removal of the A^6_g peak and a significant shift in the B^6_g peak position."

5) *I suggest the authors restructure the first paragraph of the Methods section to be four parts: (i) a one to two sentence short introduction, (ii) the 1187 atomic structures and DFT calculations, (iii)*

the development of the ML interatomic potential and its accuracy, and (iv) MD settings and simulations (NPT, NVT). While details could be found in the SI, it is still important to make clear for each part the purpose and the quantities computed.

The Methods section in the main text has been updated accordingly.

6) *In addition, I think the tables and figures in the SI should be mentioned at least once in the main text.*

This has been corrected; tables and figures in the SI are now referenced in the main text.

7) *While the writing is clear, it could still be overall polished for better readability and higher quality. For example, in the abstract, "Phase transitions of the BaZrS3 perovskite are under-explored in literature as most experimental characterization is performed at ambient conditions" could be "Phase transitions of BaZrS3 remain underexplored in literature, as most experimental characterizations of this material have been performed at ambient conditions".*

We have re-worded this sentence as suggested. In addition, we have re-worded other parts of the main text for clarity. Our changes are highlighted in the main text.

8) *Besides, I think there are some typos, in Figure 1, the Glazer notion of the Pnma phase is a+b-b-, while in the main text, it is a-a-c+.*

Thank you for spotting this. We have updated the notation in the main text so that it is consistent with the figure.

9) *Additionally, (i) I think the citation numbers in a single sentence should generally be put together, (ii) It would be better to consistently put "interatomic" before "potential", (iii) it would be more precise to say "free energies", "atomic forces", and "stress tensors", and (iv) It is not necessary to add an "NEP" before "machine learning".*

Thank you; all suggested changes have been applied throughout.

10) *"In Fig. 2, we plot properties observed and derived from MD simulations spanning 0 K to 1200 K and 200 ns." If NPT MD is performed here, it is necessary to make clear the pressure.*

This is done: "In Fig. 2, we plot properties observed and derived from MD simulations spanning 0 K to 1200 K and with no applied pressure."

11) *"All quantities are averaged over a time period corresponding to 5 K." What does "corresponding to 5 K" mean?*

This is the time period corresponding to a 5K change in temperature. The time period itself (0.8ns) has now been specified in the Figure 2 caption.

12) *"This might contribute towards the quantitative discrepancy between the experimentally measured transition at 773 K, and our predicted transition at 610 K." I guess this is a discussion of the triangle data point in Figure 3, so it is necessary to refer to Figure 3 here.*

Given the newly published data from Jaiswal et al. we have removed this scatter point from Figure 3 as we do not want to display a sub-set of the experimental data. We now make comparisons to all available experimental data in the main text only.

13) *"Both phase transitions occur above 600 K, which agrees with experimental characterisation showing BaZrS3 forms in the orthorhombic Pnma phase at ambient temperature and pressure." It seems more appropriate to use "is stable" or "exists" instead of "forms".*

This has been updated: "Both phase transitions occur above 600 K, which agrees with experimental

characterisation showing $BaZrS_3$ is stable in the orthorhombic *Pnma* phase at ambient temperature and pressure."

Reviewer 2

14) *Calorimetric Measurements: The authors have calculated the heat capacity of BaZrS3 but did not refer to experimental work on calorimetric measurements such as Ref. 56. Moreover, the authors in Ref. 56 have attributed the features in the TGA and DSC of $BaZrS_3$ as precursors leaving the sample and oxidation and not a phase transition.*

We have included an additional paragraph highlighting the oxidation processes at high temperature:

"Experimental characterisation of the high-temperature *Pm3m* phase is hindered by oxidation to form $BaSO_4$,

ZrO_2 and SO_2. Differential scanning calorimetry and thermogravimetric analysis show that BaZrS3 is stable in air up to 920 K, with complete conversion to the oxidised products at 970 K."

15) *Discussion on Figure 2: The authors claim to show the activation of modes in Figure 2 b,c. The concept of mode activation usually refers to selection rules of specific measurements. It is not clear what the authors mean by 'active'.*

Thank you for highlighting this. We have added clarification: "From 0 K to 650 K one M-mode and two R-modes are active (have a non-zero amplitude)".

16) *In the discussion on Figure 2 b,c, the authors should give references or additional explanations to the claims that mode activation correlates to the existence of specific phases.*

Thank you for highlighting this. We have now signposted the reader to an earlier paragraph and figure: "This tilt pattern is described by a+b−b− in Glazer notation and corresponds to a structure in the *Pnma* space group (see Figure 1 and the associated discussion)."

17) *Hysteresis of the First-order Transition: The authors should reference/explain the stochastic nature of MD simulations and why they result in hysteresis in their cooling runs.*

Thank-you; we have now referenced two papers which explore hysteresis in MD simulations of first-order transitions: "Hysteresis in simulations describing a first-order transition have been observed and discussed in previous studies".

18) *Typo: On page 5, at the end of the left column, there's a typo - "see Sect. for further discussion" – the section number is missing.*

This is corrected.

19) *First-order transition in Raman: It is widely acceptable that the Raman signature of tetragonal and cubic phases in perovskites are broad and very different than the orthorhombic phase. This is due to the dynamic stabilization required related to the imaginary modes. Therefore, to claim that the tetragonal and orthorhombic phases of BaZrS3 would have a similar Raman, I expect to see a calculation with no imaginary modes or at least the Gamma point mode frequencies of one.*

Thank you. This was also raised by the other reviewer; please see our response to point 4 above.

20) *Negative pressures? A general question as an experimentalist regarding the calculation and Figure 3 specifically – What's the meaning of negative pressures? Why not focus the calculations on feasible pressures that one may measure or work at? Perhaps add a discussion on the significance of phase transitions at high/negative pressures on the functionality and properties of the material*

We have now included a short discussion on the relevance of negative pressures: "Negative pressures correspond to triaxial tensile strain. Whilst this is difficult to realise experimentally, tensile strain in a plane can be produced through coherent interface formation with a suitably matched substrate. As such, Fig. 3 indicates the range of polymorphs which might be accessed through interface engineering in a device stack."
